# Systemic Availability of Human Milk Oligosaccharides in Infants and Adults: A Narrative Review

**DOI:** 10.1016/j.advnut.2025.100488

**Published:** 2025-08-06

**Authors:** Sabrina Schenk, Lars Bode, Stina Rikke Jensen, Yannik Bernd Schönknecht, Marie-Christine Simon

**Affiliations:** 1 Institute of Nutritional and Food Science, Nutrition and Microbiota, University of Bonn, Bonn, Germany; 2 Department of Pediatrics, Larsson-Rosenquist Foundation Mother-Milk-Infant Center of Research Excellence (LRF MOMI CORE), and Human Milk Institute (HMI), University of California San Diego, La Jolla, CA, United States; 3 Novonesis, Human Health Research, Hørsholm, Denmark; 4 Novonesis, HMO, Rheinbreitbach, Germany

**Keywords:** absorption, blood, excretion, human milk, human milk oligosaccharides, systemic availability, urine

## Abstract

Human milk contains many components with physiological effects beyond basic nutrition, including large quantities of structurally diverse oligosaccharides. Human milk oligosaccharides (HMOs) have been linked to health outcomes through microbiome-dependent and microbiome-independent mechanisms. To investigate the microbiome-independent effects of individual HMOs and their role in human health, it is necessary to understand their systemic availability. This narrative review focuses on the systemic availability of HMOs and summarizes studies that investigated the presence of HMOs in blood and urine following oral intake in humans. We searched PubMed using the following terms individually or in combination: human milk oligosaccharides, HMO, 2′-fucosyllactose, 3-fucosyllactose, 3′-sialyllactose, 6′-sialyllactose, difucosyllactose, lacto-*N*-tetraose, and lacto-*N*-neotetraose. The inclusion criteria were as follows: *1*) study design observational or interventional; *2*) cohort included breastfed infants, HMO–formula-fed infants or individuals taking HMO supplements; and *3*) methods defined HMO absorption/excretion and described analysis. We identified 15 human studies. They varied in design, populations (healthy infants, infants with medical indications, and adults), administration (breastfeeding, formula, and supplement), ingested dose, sampling time points, and analytical methods. HMOs were absorbed into the bloodstream and excreted in urine, as they were detected in the blood and urine of breastfed infants, infants receiving HMO–fortified formula, and adults receiving HMO supplements, demonstrating their systemic availability. Most orally ingested HMOs appeared in blood, but some structures were not absorbed. Studies also reported that blood and urine concentrations of HMOs correlated with increasing doses. Some studies showed a difference between the number of HMOs ingested and the number of oligosaccharides found in urine. Current evidence supports the systemic availability of HMOs in both infants and adults, but absorption kinetics, rates, mechanisms, and metabolic fate remain unknown. Further research investigating the systemic availability of HMOs is needed to improve our understanding of the microbiome-independent effects of HMOs on human health.

**Statements of Significance**

This narrative review of recent studies confirms that human milk oligosaccharides (HMOs) taken orally by infants and adults are absorbed into the blood and excreted in urine, indicating their systemic availability and supporting the hypothesis that physiological benefits of HMOs may not be limited to their prebiotic mode of action but may also be due to their systemic availability and effects. Key questions remain about the mechanisms of absorption, absorption kinetics, structure-specific differences, and metabolic fate of HMOs—these insights are essential for advancing our understanding of HMOs as bioactive components with systemic relevance.

## Introduction

**FIGURE 1:**
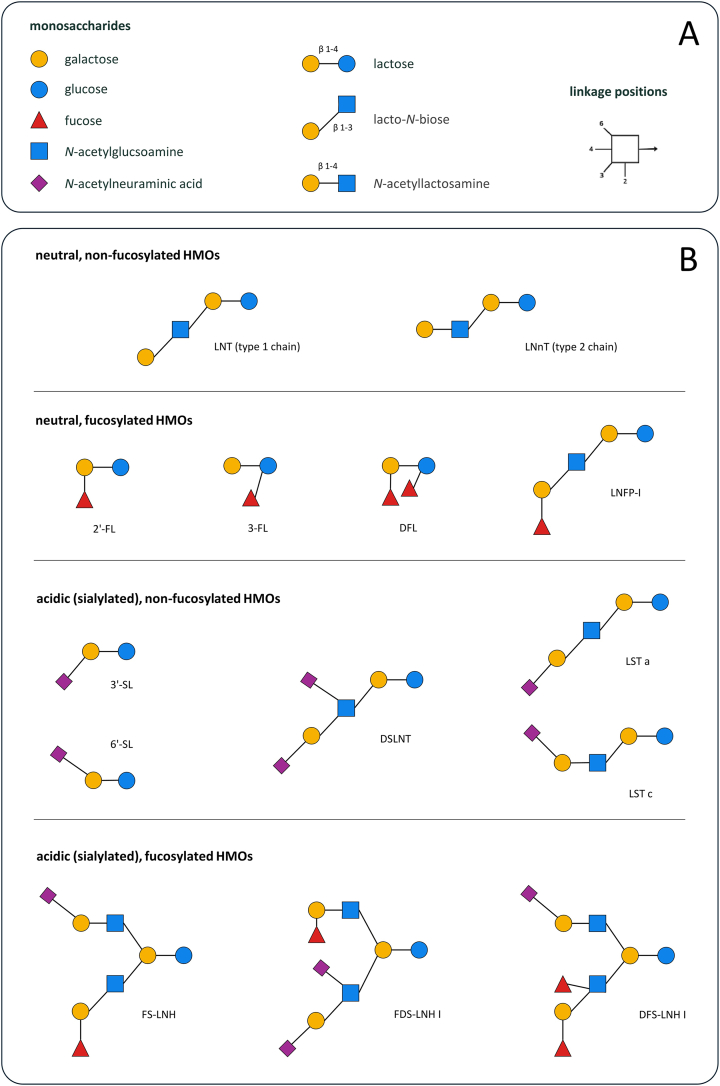
The structural diversity of human milk oligosaccharides (HMOs). (A) Monosaccharide units and linkage positions for HMO formation. Symbols for the main HMO units are explained in the key. The 3 cores that HMOs can extend are lactose, lacto-*N*-biose, and *N*-acetyllactosamine. Each unit can be linked at positions 2, 3, 4, or 6. (B) Common HMOs, categorized according to their structural characteristics. Neutral nonfucosylated HMOs are shown at the top, including lacto-*N*-tetraose (LNT) as a type 1 oligosaccharide and lacto-*N*-neotetraose (LNnT) as a type 2 oligosaccharide. Neutral fucosylated HMOs such as 2′-fucosyllactose (FL), 3-FL, difucosyllactose (DFL), and lacto-*N*-fucopentaose (LNFP)-I are shown in row 2. Acidic (sialylated) nonfucosylated HMOs including 3′-sialyllactose (SL), 6′-SL, disialyl-lacto-*N*-tetraose (DSLNT), sialyllacto-*N*-tetraose (LST)a and LSTc are shown in row 3. Acidic (sialylated) fucosylated HMOs, including fucosyl-sialyl-lacto-*N*-hexaose (FS-LNH), fucosyl-difucosyl-sialyl-lacto-*N*-hexaose (FDS-LNH) I, and difucosyl-sialyl-lacto-*N*-hexaose (DFS-LNH) I are shown in the bottom row.

Human milk is a complex biological system that provides macronutrients and micronutrients, as well as other bioactive compounds that exert physiological effects [1]. A special feature of human milk is the high content and structural diversity of human milk oligosaccharides (HMOs). These are complex glycans composed of 5 monosaccharide building blocks, namely glucose (Glc), galactose (Gal), *N*-acetylglucosamine (GlcNAc), fucose, and sialic acid, with *N*-acetylneuraminic acid being the most predominant form of sialic acid. Different arrangements and combinations of these building blocks result in >150 structurally distinct HMOs. All HMOs carry lactose (Galβ1–4Glc) at the reducing end, which can be elongated by adding β1–3-linked lacto-*N*-biose (Galβ1–3GlcNAc) or β1–4-linked *N*-acetyllactosamine (Galβ1–4GlcNAc). The addition of lacto-*N*-biose forms a type 1 chain (type 1 oligosaccharide) such as lacto-*N*-tetraose (LNT), whereas the addition of *N*-acetyllactosamine leads to the formation of a type 2 chain (type 2 oligosaccharide) such as lacto-*N*-neotetraose (LNnT). Furthermore, lactose or the elongated oligosaccharide chains can be modified by the addition of fucose (α1-2, α1-3, or α1-4 linkages) to form HMOs such as 2′-fucosyllactose (FL), 3-FL, and difucosyllactose, and/or by the addition of sialic acid (α2-3 or α2-6 linkages) to form HMOs such as 3′-sialyllactose (SL) and 6′-SL. Based on these structural modifications, HMOs can be divided into 4 main groups: neutral nonfucosylated, neutral fucosylated, acidic (sialylated) nonfucosylated, and acidic (sialylated) fucosylated [2] (Figure 1).

In vitro work showed that HMOs are resistant to hydrolysis by salivary, pancreatic, and brush border enzymes, as well as enzymes at low gastric pH [3,4]. Therefore, HMOs are considered to be mostly resistant to digestion and thus reach the colon undigested, where they are available for selective utilization by bacteria [5,6]. Accordingly, HMOs act not only as prebiotics that modulate the gut microbiome but also as antimicrobial agents with bacteriostatic, bacteriocidal, or antiadhesive effects against intestinal bacteria and pathogens such as viruses and protozoan parasites [7,8]. Moreover, HMOs may exert microbe-independent effects by modulating cell recognition and cell signaling [9]. These include interactions with immune cells, thereby influencing immune system development and immune responses, maturation of the intestinal glycocalyx [10], neurodevelopment, and cognitive functions [11,12]. For such systemic, microbiome-independent effects to occur, HMOs must be absorbed into the bloodstream, making them systemically available to tissues and organs other than the gastrointestinal tract.

In nutritional science, bioavailability is defined as the proportion of intake that can be absorbed by the intestine and made available for physiological and metabolic functions, or for storage [13]. The concept of bioavailability includes availability for absorption (or bioaccessibility), absorption, tissue distribution, and bioactivity [14]. The storage and utilization of HMOs is a key research topic, and the first step is to characterize their systemic availability, which is defined by absorption kinetics and excretion.

Research on individual HMOs has been limited by restricted accessibility, but recent technological advances now allow the production of some oligosaccharides that are chemically and structurally identical to those in human milk. Several HMOs can be manufactured by microbial fermentation [15], enzymatic conversion [16], or other technologies [17] in sufficient quantities for clinical studies and application purposes [18].

Understanding the systemic availability of HMOs, including the extent of absorption, factors that influence absorption, and their ultimate fate, is necessary to understand their microbiome-independent effects and potential role in human health. This review focuses on the systemic availability of HMOs in infants and adults, summarizing studies that investigated the presence of HMOs in blood and urine following oral intake.

## Methods

In October 2024, we conducted a literature search on PubMed using the following search terms individually or in combination: human milk oligosaccharides, HMO, 2′-fucosyllactose, 3-fucosyllactose, 3′-sialyllactose, 6′-sialyllactose, difucosyllactose, LNT, and lacto-*N*-neotetraose. We used article titles and abstracts to identify studies that met 3 criteria: *1*) the study design was observational or interventional; *2*) the cohort included breastfed infants, HMO–formula-fed infants, and/or individuals who had taken HMO supplements; and *3*) the methods defined HMO absorption/excretion and described the analysis. To ensure the search was complete, we also reviewed the selected articles, as well as articles in our collections and related bibliographies using the “see all similar articles” feature in PubMed. For each result that met our criteria, we extracted the author, sample size, HMO administration route (source), analytical and sampling methods, blood and urine analysis, and key findings. We selected publications containing original research on humans. We studied the full text and references, and if the latter included additional eligible articles, those were also included.

## Results

We identified 15 studies that met our inclusion criteria, 12 in infants and 3 in adults, comprising 8 observational and 7 interventional studies. Overall, the included studies were hihly heterogeneous in terms of study design, populations (healthy infants, infants with medical conditions, and adults), administration (breastfeeding, formula, and supplement), ingested dose, sampling time points, and analytical methods.

Of the 12 studies in infants, 8 included healthy infants, 1 featured infants with medical conditions, and 3 did not provide health information. Five studies focused on term infants, 3 on preterm infants, and 4 did not report gestational age at birth. For the 3 studies in adults, 1 considered individuals with irritable bowel syndrome; 1 considered individuals with glucosamine (UDP-*N*-acetyl)-2-epimerase/N-acetylmannosamine kinase (GNE) myopathy, a muscle disorder; and 1 focused on healthy but overweight individuals.

Seven studies measured HMOs in blood and 11 in urine, with 3 looking at both. HMOs were detected in 11 studies after breastfeeding, in 2 after feeding HMO-fortified infant formula (1 study considered both), and in 3 studies providing individual HMOs as supplements. HMO absorption and excretion were stated as individual concentrations (eg, in milligrams per liter) or relative abundances (percentage of total HMOs detected) in the blood and urine samples, respectively (Supplemental Table 1). The studies differed in design, population, HMO administration (breastfeeding, formula, or supplement), analytical methods (quantitative or semiquantitative), sampling time points, orally ingested dosage, and type of analysis (targeted or untargeted). The studies are summarized in Table 1 [[19], [20], [21], [22], [23], [24], [25], [26], [27], [28], [29], [30], [31], [32], [33]], and the specific oligosaccharides that were analyzed and detected are listed in Table 2.

**TABLE 1:** Summary of studies on HMO absorption and excretion in humans.

Study	Population	Investigation	Administration	Sampling time points	Analytical methods	Key findings
Radzanowski et al., 2013 [19]	55 healthy infants	2′-FL, 3-FL, 3′-SL and 6′-SL concentrations in infant blood	Exclusively breastfed	Not stated	LC-MS/MS, HPAEC	2′-FL, 3′-SL, and 6′-SL detected in blood
Goehring et al., 2014 [20]	35 healthy term infants	2′-FL, 3-FL, 6′-SL, 6′-SLN^1^, LNnT, LNFP I, LNFP II, and LNFP III concentrations in infant blood and urine	Exclusively breastfed (*n* = 18) Non–HMO-fortified formula-fed (*n* = 17)	Blood (42 d of age) Urine (14 d of age)	GC/MS, LC-MS/MS, HPLC	Breastfed infants: - 2′-FL, 3-FL, and LNnT detected in blood - 2′-FL, 3-FL, LNnT, LNFP I, LNFP II, LNFP III, 6′-SL, and 6′-SLN^1^ detected in urine Non–HMO-fortified formula-fed infants: - HMOs not detected in blood or urine, except for 6′-SLN^1^ in urine
Ruhaak et al., 2014 [22]	13 term infants hospitalized due to congenital heart disease	Abundance of oligosaccharides in infant blood	Partially breastfed (infants received a mixed feeding regimen, including human milk and non–HMO-fortified formula) (*n* = 9) exclusively non–HMO-fortified formula-fed (*n* = 4)	Blood (<30 d of age)	nLC-PGC-chip-TOF-MS	2′-FL, LNT, LNnT, LNFP III, DFL, 3′-SL, 6′-SL, LST, 3′-SLN^1^, and 6′-SLN^1^ detected in blood of all infants 2′-FL, LNT, LNnT, LNFP III, DFL, 6′-SL, LST, 3′-SLN^1^, and 6′-SLN^1^ detected in exclusively formula-fed infants but at lower abundance than in partially breastfed infants 2'-FL and LNFP III stated as nearly absent in plasma from exclusively formula-fed infants
Marriage et al., 2015 [21]	304 healthy term infants	2′-FL concentration in infant blood and urine	HM: exclusively breastfed (*n* = 83) EF1: formula-fed with 0.2 g/L 2′-FL (*n* = 70) EF2: formula-fed with 1.0 g/L 2′-FL (*n* = 72) CF: non–HMO-fortified formula-fed (*n* = 79)	Blood (42 d, 119 d of age) Urine (42 d, 119 d of age)	LC-MS/MS, UHPLC, electrospray ionization	Breastfed and 2′-FL formula-fed infants (*n* = 225): - 2′-FL detected in blood (HM>EF2>EF1) - 2′-FL detected in urine (HM, EF2>EF1) - comparable relative absorption and excretion of 2′-FL among the groups fed 2′-FL Non–HMO-fortified formula-fed infants: - HMOs not detected in blood or urine
Rudloff et al., 1996 [32]	18 healthy preterm infants	Abundance of oligosaccharides in infant urine	Exclusively breastfed (*n* = 9) Non–HMO-fortified formula-fed (*n* = 9)	Not stated	FAB-MS, HPAE-PAD	Oligosaccharides detected in urine of breastfed and formula-fed infants but structures not all named individually Exclusively breastfed infants: - oligosaccharides detected in urine including 3-FL, DFL, 3′-SL, 6′-SL, LNT, fucosylated and sialylated derivatives of LNT, LNFP I and II, and complex oligosaccharides such as lacto-*N*-hexaose, difucosyllacto-*N*-hexaose, and sialyllacto-*N*-tetraose - more complex oligosaccharide patterns in breastfed infants than those in formula-fed infants Non–HMO-fortified formula-fed infants: - oligosaccharides detected in urine including 3-FL, DFL, LNT, 3′-SL, 6′-SL, and sialyllacto-*N*-tetraose
Chaturvedi et al., 2001 [33]	32 healthy infants	Abundance of oligosaccharides in infant urine	Exclusively breastfed (*n* = 16) Non–HMO-fortified formula-fed (*n* = 16)	Not stated	HPLC	Oligosaccharides detected in urine of all infants Higher oligosaccharide concentrations in urine of breastfed infants than in formula-fed infants
De Leoz et al., 2013 [31]	Healthy preterm infants (number not stated)	Abundance of 75 oligosaccharides in infant urine	Fortified human milk	Not stated	nLC-chip-TOF-MS, MALDI FT-ICR MS	17 oligosaccharides^1^ detected in urine
Dotz et al., 2015 [30]	10 healthy term infants	Abundance of oligosaccharides in infant urine	Exclusively breastfed	36-h urine samples	MALDI-TOF MS, HPAEC-PAD	>70 intact oligosaccharides^1^ detected in urine, with major oligosaccharides in infant urine: LNDFH I, 3-FL, LNFP II, DFL, 2′-FL, LNnT, LNT, and LNFP I
Underwood et al., 2015 [29]	14 preterm infants	Abundance of 60 oligosaccharides in infant urine	Exclusively breastfed	Not stated	MS	56 oligosaccharides^1^ detected in urine
Obermeier et al., 1999 [27]	1 infant	Abundance of ^13^C-labeled HMOs in infant urine	Exclusively breastfed ^13^C-galactose bolus for breastfeeding mother	24-h urine sample	IRMS, characterization by FAB-MS, HPAEC-PAD	^13^C-labeled HMO structures appeared in urine
Rudloff et al., 2012 [26]	10 infants	Abundance of ^13^C-labeled HMOs in infant urine	Exclusively breastfed infants ^13^C-galactose bolus for breastfeeding mother	36-h urine samples	IRMS, characterization by FAB-MS	^13^C-labeled HMO structures appeared in urine
Dotz et al., 2014 [28]	10 healthy term infants	Abundance of ^13^C-labeled HMOs in infant urine	Exclusively breastfed infants ^13^C-galactose bolus for breastfeeding mother	36-h urine samples	MALDI-TOF MS	^13^C-labeled HMO structures appeared in urine
Iribarren et al., 2021 [23]	58 adults with IBS (aged 19–73 y)	Abundance of 2′-FL, LNnT in adult blood and urine	4-wk supplementation: 2′-FL + LNnT (ratio 4:1), 10 g/d (*n* = 19) 2′-FL + LNnT (ratio 4:1), 5 g/d (*n* = 20) Glucose (placebo), 5 g/d (*n* = 19)	Blood (0 d, 28 d of study participation) Urine (0 d, 28 d of study participation)	LC-MS, UPLC	2′-FL detected in blood and urine in each intervention group Significant increase only in blood and urine concentrations of 2′-FL in groups supplementing HMOs, but not in placebo group LNnT not detected in any samples
Park et al., 2023 [25]	10 adults with GNE myopathy (aged 18–70 y)	6′-SL concentrations in adult blood	Single-dose supplementation: 6′-SL 3 or 6 g	Blood (0.5, 1, 1.5, 2, 4, 6, 8, and 24 h after dosing)	LC-MS/MS	6′-SL detected in blood Orally administered 6′-SL was rapidly absorbed and its blood concentration increased in a dose-dependent manner within 0.5–1 h
Ko et al., 2024 [24]	41 overweight adults (aged 18–65 y with BMI 25–40 kg/m^2^ or with body fat > 30%)	2′-FL concentrations in adult blood	12-wk supplementation: 2′-FL, 3 g/d (*n* = 21) Maltodextrin (placebo), 3 g/d (*n* = 20)	Blood (after 6 and 12 wk of supplementation)	Quantitative assay for 2′-FL (1-pot reaction with α1,2-fucosidase and l-fucose dehydrogenase)	Increase in blood concentrations of 2′-FL following ingestion 2*'*-FL detected in blood of placebo group, but at lower concentrations than in group supplemented with 2*'*-FL

Abbreviations: CF, control formula; DFL, lacto-*N*-difucohexaose; DFLNH, difucosyllacto-*N*-hexaose; EF, experimental formula; FAB-MS, fast atom bombardment-MS; FL, fucosyllactose; GC, gas chromatography; GNE, glucosamine (UDP-*N*-acetyl)-2-epimerase/*N*-acetylmannosamine kinase; HM, human milk; HMO, human milk oligosaccharide; HPAEC, high-performance anion-exchange chromatography; HPAE-PAD, high-performance anion-exchange chromatography with pulsed amperometric detection; IBS, irritable bowel syndrome; IRMS, isotope ratio MS; LC-MS/MS, liquid chromatography with tandem MS; LNDFH, lacto-*N*-difucotetraose; LNFP, lacto-*N*-fucopentaose; LNnT, lacto-*N*-neotetraose; LNT, lacto-*N*-tetraose; LST, sialyllacto-*N*-tetraose; MALDI FT-ICR MS, matrix-assisted laser desorption/ionization Fourier transform-ion cyclotron resonance MS; MALDI-TOF MS, matrix-assisted laser desorption/ionization time-of-flight-MS; MFLNH, monofucosyllacto-*N*-hexaose; nHPLC-PGC-chip-TOF-MS, nano–high-performance liquid chromatography–chip time-of-flight MS; nLC-PGC-chip-TOF-MS, nanoliquid chromatography–chip time-of-flight MS; SL, sialyllactose; SLN, sialyllactosamine; UHPLC, ultrahigh-performance liquid chromatography; UPLC, ultraperformance liquid chromatography.

1 Study referred to detected structures as HMOs, but debatable whether all structures are HMOs or structurally similar glycans.

**TABLE 2:** Oligosaccharides analyzed and detected in human blood and urine samples.

Study	Oligosaccharide*s* analyzed		Oligosaccharide*s* detected	
	Blood	Urine	Blood	Urine
Radzanowski et al., 2013 [19]	4 structures analyzed: 3′-SL, 6′-SL, 2′-FL, and 3-FL	Not analyzed	3 structures detected: 2′-FL, 3′-SL, and 6′-SL	Not analyzed
Goehring et al., 2014 [20]	8 structures analyzed: 2′-FL, 3-FL, LNnT, LNFP I, LNFP II, LNFP III, 6′-SL, and 6′-SLN	8 structures analyzed: 2′-FL, 3-FL, LNnT, LNFP I, LNFP II, LNFP III, 6′-SL, and 6′-SLN	3 structures detected: 2′-FL, 3-FL, and LNnT	8 structures detected: 2′-FL, 3-FL, LNnT, LNFP I, LNFP II, LNFP III, 6′-SL, and 6′-SLN
Ruhaak et al., 2014 [22]	Untargeted	Not analyzed	10 structures detected: LNT, LNnT, LNFP III, DFL, 2′-FL, 3′-SL, 6′-SL, LST, 3′-SLN, and 6′-SLN	Not analyzed
Marriage et al., 2015 [21]	1 structure analyzed: 2′-FL	1 structure analyzed: 2′-FL	1 structure detected: 2′-FL	1 structure detected: 2′-FL
Rudloff et al., 1996 [32]	Not analyzed	Untargeted	Not analyzed	Individual structures not all specified, but among others 3-FL, DFL, 3′-SL, 6′-SL, LNT, LNFP I, and LNFP II
Chaturvedi et al., 2001 [33]	Not analyzed	Untargeted	Not analyzed	Individual structures not all specified, but among others 3-FL, 2′-FL, LNF I, and LDFH I
De Leoz et al., 2013 [31]	Not analyzed	75 structures analyzed: 3-FL, 2′-FL, 3′-SL, 6′-SL, DFL, 3′-SLN, 6′-SLN, LNT, LNnT, 3′-sLe, LNFP I, LNFP II, LNFP III, LNFP V, LSTa, LSTb, 3011a, LNDFH I, LNDFH II, p-LNH, LNH, LNnH, SFLNnT, A-hepta, MFpLNH IV, 4120a (FS-para-LNnH I), MFLNH I, MFLNH III, IFLNH I, IFLNH III, DSLNT, 4021a (S-LNH II), 4021b (S-para-LNnH), MSLNnH, S-LNH, DFpLNH II, DFLNHa, DFLNHb, DFLNHc, 4121a (FS-para-LNnH I), 4121b (FS-para-LNnH II), MSMFLNnH, MSMFLNH I, FS-LNH I, FS-LNH II, FS-LNH III, TFLNH, 4320a, F-LNO, 5130a (F-iso LNO), 5130b (F-LNO II), 5130c (F-LNO III), MSDFLNnH, FS-LNH, 5031a (S-LNO), DFLNO I, DFLNnO II, 5230a (DF-is-LNO VII), DFLNnO I or DFLNO II, 5230b (DF-LNnO III), 5131a (FS-LNO), TFiLNO, 5330a, 5331a, 5231a, 5231b, tetra-iso-LNO, 6041a (sLNnD), 6140a (F-LND II), 6240a, 6340a (TriF-LND V), 6340b (TriF-LND VI), 6340c (TriF-LND VII), and 6440a (TetraF-LND III)	Not analyzed	17 structures detected: 3′-SL, DFL, 3′-SLN, 6′-SLN, LNT, LNnT, 3′-sLe, LNFP II, LNFP III, LSTb, LNDFH I, LNH, SFLNnT, A-hepta, DFLNHa, MSMFLNH I, and 5130a (F-iso LNO)
Dotz et al., 2015 [30]	Not analyzed	Untargeted	Not analyzed	>70 structures detected (individual structures not all specified, but among others LNDFH I, 3-FL, LNFP II, DFL, 2′-FL, LNnT, LNT, and LNFP I)
Underwood et al., 2015 [29]	Not analyzed	60 structures analyzed: 3-FL, 2′-FL, DFL, LNT/LNnT, LNFP I, LNFP II, LNFP III, LNFP V, LNDFH I/LNDFH II, LNH, LNnH, p-LNH, A-hepta, MFpLNH IV, 4120a (FS-para-LNnH I), MFLNH I, MFLNH III, IFLNH I, IFLNH III, DFpLNH II, DFLNHa, DFLNHb, DFLNHc, TFLNH, 5130a (F-iso LNO), 5130b (F-LNO II), 5130c (F-LNO III), F-LNO, DFLNO I, DFLNO II, DFLNnO I, DFLNnO II, 5230a (DF-is-LNO VII), 5230b (DF-LNnO III), TFiLNO, 5330a, 6140a (F-LND II), 3′-SL, 6′-SL, 3′-SLN, 6′-SLN, 3′-sLe, LST a/b/c, F-LST c, DSLNT, S-LNH, S-LNnH II, 4021a (S-LNH II), 4021b (S-para-LNnH), 4121a (FS-para-LNnH I), 4121b (FS-para-LNnH II), FS-LNH, FS-LNH I, FS-LNH III, FS-LNnH I, FS-LNH II, DFS-LNH, 5031a (S-LNO), 5131a (FS-LNO), and 6041a (sLNnD)	Not analyzed	56 structures detected: 3-FL, 2′-FL, DFL, LNT/LNnT, LNFP I, LNFP II, LNFP III, LNFP V, LNDFH I/LNDFH II, LNH, LNnH, p-LNH, A-hepta, MFpLNH IV, 4120a (FS-para-LNnH I), MFLNH I, MFLNH III, IFLNH I, IFLNH III, DFpLNH II, DFLNHa, DFLNHb, DFLNHc, TFLNH, 5130a (F-iso LNO), 5130b (F-LNO II), 5130c (F-LNO III), F-LNO, DFLNO I, DFLNO II, DFLNnO I, DFLNnO II, 5230a (DF-is-LNO VII), 5230b (DF-LNnO III), 3′-SL, 6′-SL, 3′-SLN, 6′-SLN, 3′-sLe, LSTa/b/c, F-LSTc, DSLNT, S-LNH, S-LNnH II, 4021a (S-LNH II), 4021b (S-para-LNnH), 4121a (FS-para-LNnH I), 4121b (FS-para-LNnH II), FS-LNH, FS-LNH I, FS-LNH III, FS-LNnH I, FS-LNH II, DFS-LNH, 5031a (S-LNO), and 5131a (FS-LNO)
Obermeier et al., 1999 [27]	Not analyzed	^13^C-labeled HMOs (individual structures not specified)	Not analyzed	^13^C-labeled HMOs (individual structures not specified)
Rudloff et al., 2012 [26]	Not analyzed	^13^C-labeled HMOs (individual structures not specified)	Not analyzed	^13^C-labeled HMOs (individual structures not specified)
Dotz et al., 2014 [28]	Not analyzed	^13^C-labeled HMOs (individual structures not specified)	Not analyzed	^13^C-labeled HMOs (individual structures not specified)
Iribarren et al., 2021 [23]	2 structures analyzed: 2′-FL and LNnT	2 structures analyzed: 2′-FL and LNnT	1 structure detected: 2′-FL	1 structure detected: 2′-FL
Park et al., 2023 [25]	1 structure analyzed: 6′-SL	Not analyzed	1 structure detected: 6′-SL	Not analyzed
Ko et al., 2024 [24]	1 structure analyzed: 2′-FL	Not analyzed	1 structure detected: 2′-FL	Not analyzed

Abbreviations: DFL, lacto-*N*-difucohexaose; DFLNH, difucosyllacto-*N*-hexaose; FL, fucosyllactose; HMO, human milk oligosaccharide; LNDFH, lacto-*N*-difucotetraose; LNFP, lacto-*N*-fucopentaose; LNnT, lacto-*N*-neotetraose; LNT, lacto-*N*-tetraose; LST, sialyllacto-*N*-tetraose; MFLNH, monofucosyllacto-*N*-hexaose; SL, sialyllactose; SLN, sialyllactosamine.

### Oligosaccharides in blood

Seven studies analyzed HMOs in the blood following oral intake, 4 in infants and 3 in adults. HMOs were detected in all cases (Table 1).

#### Oligosaccharides in infant blood

One study analyzed 2′-FL, 3′-SL, and 6′-SL in the blood of 55 healthy, exclusively breastfed infants. The blood concentrations of 2′-FL (0–2.25 g/L; this is likely to be a unit error in the publication and the actual reading was probably 0–2.25 mg/L rather than grams per liter), 3′-SL (0.10–0.78 mg/L), and 6′-SL (0.05–0.68 mg/L) were lower than the corresponding concentrations in human milk [19]. Another study detected 2′-FL (1.16 ± 0.29 mg/L), 3-FL, and LNnT in the blood of 18 healthy, term, exclusively breastfed infants. HMOs were absent from blood samples of 17 healthy, term, formula-fed infants (non–HMO-fortified formula), except for 2′-FL, which was present but only at negligible concentrations (<0.03 mg/L) [20]. The same group analyzed 2′-FL concentrations in the blood of 304 healthy, term infants, 83 were exclusively breastfed, 142 were fed with 2′-FL–fortified formula at 0.2 or 1.0 g/L, and 79 were fed non–HMO-fortified formula. The presence of 2′-FL was confirmed in blood samples of all study groups receiving 2′-FL, but concentrations differed between study groups in the order of ingested intake and were highest in breastfed infants, followed in turn by infants receiving formula containing 1.0 and 0.2 g/L of 2′-FL. 2′-FL was not detected in the blood circulation of infants fed non–HMO-fortified formula [21]. Relative absorption rates (proportion of ingested amount) ranged from 0.05% to 0.1% [20,21]. Further investigations detected various oligosaccharide structures in the blood of term infants hospitalized with congenital heart disease. A subgroup of 9 infants received a mixed feeding regimen, including human milk and non–HMO-fortified formula, whereas 4 infants were exclusively fed on non–HMO-fortified formula. The concentrations of LNT and 6′-SL were significantly lower in the exclusively formula-fed group (LNT 0.007 mg/L; 6′-SL 0.023 mg/L) than those of the breastfed group (LNT 0.077 mg/L; 6′-SL 0.15 mg/L) [22].

#### Oligosaccharides in adult blood

Fifty-eight adults with irritable bowel syndrome symptoms (aged 19–73 y) were provided with a 4:1 mixture of 2′-FL and LNnT at doses of 5 and 10 g/d for 4 wk. The concentrations of 2′-FL in the blood increased significantly (semiquantitative measurements) after supplementation, whereas LNnT was not detected in the blood at baseline or after the supplementation period [23]. In another study of 41 overweight adults (aged 18–65 y with BMI of 25–40 kg/m^2^ or with body fat of >30%), 21 participants received supplements of 3 g 2′-FL/d for 12 wk, whereas 20 participants received a placebo. The supplement increased the concentration of 2′-FL in the blood, but 2′-FL was also present at lower concentrations in the blood of the placebo group [24]. An additional study provided 3 or 6 g supplements of 6′-SL to 10 adults with GNE myopathy (aged 18–70 y) and measured blood concentrations ≤24 h after intake. The concentrations of 6′-SL in the blood increased significantly 30 min after administration, reaching maximum concentrations after 30 min in the low-dose group (0.139 mg/L) and after 1 h in the high-dose group (0.399 mg/L), confirming that the increases in 6′-SL were dependent on the oral dose [25].

### Oligosaccharides in urine

Eleven studies analyzed HMOs in the urine after oral intake, 10 in infants and 1 in adults. Nine of the publications involving infants focused on breastfeeding, and 1 included both a breastfed cohort and a cohort receiving HMO-fortified formula [21]. All studies detected oligosaccharides in urine after breastfeeding or supplementation (Table 1).

#### Oligosaccharides in infant urine

Breastfeeding females received ^13^C-labeled galactose in 3 studies, and in all cases, the label was incorporated into HMOs that were subsequently detected in the urine of breastfed infants [[26], [27], [28]]. Some studies analyzed several oligosaccharides in human milk and correlated those with the profiles in urine samples of breastfed infants. One study included 14 preterm, exclusively breastfed infants and quantified a diverse group of oligosaccharides, 56 of which were detected in the urine samples [29]. Another group studied 10 healthy, term, exclusively breastfed infants and found >70 oligosaccharides in urine samples [30]. The number of oligosaccharides detected in human milk and urine differed, with some being present in urine but not milk [20,26,[29], [30], [31], [32]]. A further study looked at the abundance of 75 oligosaccharides in the urine of healthy, preterm infants provided with human milk containing different human milk fortifiers. A variety of oligosaccharides was detected in urine, including neutral, nonfucosylated, fucosylated, and sialylated structures [31]. Another study analyzed oligosaccharide profiles in the urine of 16 healthy, exclusively breastfed infants and 16 fed on non–HMO-fortified formula, revealing that oligosaccharides were only detected in the urine of breastfed infants [33]. Similarly, a study analyzing oligosaccharides in the urine of 18 breastfed infants and 17 provided with non–HMO-fortified formula found that multiple oligosaccharides were present in the urine of breastfed infants but not in the formula-fed group. The concentrations of 2′-FL and 6′-SL in urine were ∼4% of the corresponding concentration in human milk [20]. Another study compared the renal excretion of oligosaccharides in healthy, preterm infants fed on human milk or non–HMO-fortified formula. The quantitative urinary excretion of oligosaccharides was similar in both cohorts, with neutral sugars derived from complex oligosaccharides excreted at a rate of 3.8 ± 2.1 mg/kg per day in breastfed infants and 2.9 ± 0.9 mg/kg per day in formula-fed infants. In this study, no distinction was made between individual oligosaccharide structures, but all neutral sugars were quantified. Greater diversity and more complex oligosaccharides such as LNT, lacto-*N*-fucopentaose (LNFP) I, and LNFP II were found only in breastfed infants [32]. Another study analyzed HMOs in the urine of 221 healthy, term infants receiving formula supplemented with 0.2 or 1.0 g/L 2′-FL or a non–HMO-fortified formula and of 83 healthy, term, exclusively breastfed infants. Breastfed infants and infants that received formula with 1.0 g/L 2′-FL showed similar concentrations of 2′-FL in urine, whereas concentrations were significantly lower in infants receiving formula with 0.2 g/L 2′-FL. No 2′-FL was detected in the urine samples of the non–HMO-fortified formula group [21].

#### Oligosaccharides in adult urine

In the single adult study, we considered, supplementation with a 4:1 ratio of 2′-FL and LNnT at 5 or 10 g/d for 4 wk resulted in a significant increase in the concentrations of 2′-FL in urine (semiquantitative measurements), but no LNnT was detected [23].

### Number of structures analyzed and detected in blood and urine

The HMO structures analyzed and detected in the 15 studies are listed in Table 2. The most widely studied HMO was 2′-FL, which was investigated in 9 studies and identified in both blood [[19], [20], [21], [22], [23], [24]] and urine after oral intake [20,21,23,29,31,33]. Several studies detected other HMOs in blood and urine samples after oral ingestion, including 3-FL, 3′-SL, 6*'*-SL, LNT, LNFP I, and LNFP II.

### Analytical methods

The studies used different analytical techniques to assess the oligosaccharide structures in biological samples, with HPLC and high-performance anion-exchange chromatography, the most frequently used separation methods, and MS, the most frequently used detection method. Separation and detection techniques were often combined (Table 1). Eight studies investigated HMO absorption and excretion by the targeted analysis of specific HMO structures [[19], [20], [21],[23], [24], [25],^29^,^31^], whereas 7 used untargeted analysis [22,[26], [27], [28],^30^,^32^,^33^], of which 3 used C^13^-labeling to detect HMOs [[26], [27], [28]].

## Discussion

This narrative review summarizes and maps studies on the systemic availability of HMOs after oral intake in humans. We identified 15 publications reporting data in different populations, mostly not only infants but also adults. These studies showed that HMOs, whether ingested orally in human milk, HMO-fortified infant formula or as dietary supplements, can be detected in blood and urine, indicating their systemic availability.

Some studies suggest that blood and urine concentrations of HMOs correlate with the amount of HMOs ingested [20,21,25]. Higher doses of HMOs may therefore lead to higher concentrations of HMOs in blood and urine [21]. In infants, HMO concentrations in blood and urine correlated with corresponding concentrations in human milk [20]. Likewise, formula containing 1.0 g/L 2′-FL resulted in higher blood concentrations of 2′-FL than formula containing 0.2 g/L 2′-FL [21]. Similar results were reported in adults receiving 3 or 6 g of 6′-SL in a single dose, with blood concentrations of 6′-SL increasing in a dose-dependent manner [25]. These findings support a potential dose-dependent relationship between HMO intake and systemic availability. Additional support comes from a preprint study (not included in this review, published after submission), which found that adults receiving 1 or 5 g of 2′-FL daily for 6 wk had higher concentrations of 2′-FL in both blood and urine in the high-dose group [34]. This dose dependency is consistent with findings from studies in breastfed infants, where 2′-FL, the most abundant HMO in the milk of secretor mothers, is also the most frequently detected HMO in systemic circulation [35].

The reported data do not allow the precise measurement of the rate of absorption of HMOs into the blood. With the exception of one study in adults using controlled doses of 3 and 6 g of 6′-SL, none controlled the amount of HMOs ingested. Particularly, in infant studies, the amount of HMOs ingested cannot be quantified accurately because the volume of human milk or HMO-fortified formula consumed was not reported, and the concentration of HMOs in human milk was not measured. Consequently, the percentage absorbed can only be estimated.

HMOs of varying molecular sizes and structural groups have been detected in the blood and urine of breastfed infants [22,31] and appeared largely metabolically unmodified in the urine [26,27,36]. However, the metabolic fate of HMOs has not yet been investigated in detail and current data do not reveal whether HMOs reach systemic circulation intact or undergo metabolization prior to detection in blood and urine. In particular, more complex HMO structures may be subject to microbial degradation in the gut before absorption or hepatic degradation after absorption [5]. Further research is needed to understand these processes.

It may be speculated that the systemic availability of HMOs in infants may be attributable in part to greater intestinal permeability during early life. The neonatal intestinal barrier is more permeable at birth and undergoes maturation during the first months of life [37], potentially allowing larger molecules, including HMOs, to more readily cross into the bloodstream [38]. However, studies in adults have also shown the presence of HMOs in blood and urine after supplementation [[23], [24], [25]]. Whether infants absorb HMOs more efficiently than adults remains unclear because there are no standardized studies to allow proper comparisons. Current evidence indicates that HMO absorption occurs in both infants and adults. In addition, Ruhaak et al. [22] stated that sialylated HMOs, such as 3′-SL and 6′-SL, as well as sialylated glycans, such as 3′-sialyllactosamine (SLN), 6′-SLN were present in the blood of partially breastfed and exclusively non–HMO-fortified formula-fed infants (hospitalized due to congenital heart disease). These may derive from cow milk–based formulas, although they are present at low concentrations in cow milk and therefore in formula [39]. Compared with human milk, cow milk contains a higher abundance of sialylated and a lower abundance of fucosylated oligosaccharides [8]. It is possible that these hospitalized infants had altered gut permeability due to chronic illness, antibiotic use or surgery. This could potentially make them more likely to absorb cow oligosaccharides at higher concentrations than healthy infants.

The cellular and molecular mechanisms underlying HMO absorption remain largely unknown. Current knowledge suggests that HMOs reach the small intestine largely intact because they are resistant to gastric acidity and digestion by pancreatic and brush border enzymes [3,4]. However, there are few mechanistic studies dealing with how HMOs traverse the intestinal epithelium. One study using a Caco-2 monolayer model in vitro suggests that neutral HMOs can cross the intestinal barrier by cellular transcytosis. Specifically, 1%–1.5% of apically applied oligosaccharides crossed the monolayer, but LNT was transported from the apical to the basolateral compartment in significantly higher amounts than the acidic HMO 6′-SL [40]. However, Caco-2 cell models are no longer considered state-of-the-art, and more advanced in vitro systems are available to better mimic physiological conditions, enabling detailed investigations of oligosaccharide absorption in different segments of the intestine [41]. Future studies should therefore examine the absorption mechanisms of a broader range of HMOs using these physiologically more relevant models.

Our findings also highlight a significant gap in the evidence regarding the kinetics of HMO absorption, with only a single study considering the absorption kinetics of 6′-SL in adults with GNE myopathy [25]. These adults received a single dose of 6′-SL and blood samples were collected at multiple time points (0.5, 1, 1.5, 2, 4, 6, 8, and 24 h after dosing). Rapid absorption of 6′-SL was observed, with peak concentrations (*C*_max_) reached after 30 min in the low-dose group and after 1 h in the high-dose group. The mean *C*_max_ was higher in the high-dose group (0.399 mg/L) than that in the low-dose group (0.139 mg/L) [25]. Further clinical studies are needed to investigate the absorption kinetics of additional HMOs under controlled conditions, enabling the determination of pharmacokinetic parameters such as *C*_max_, *t*_max_ (time to peak concentration), and clearance rates.

The available data on HMOs in urine highlight certain limitations. Glycans naturally occur in human urine as metabolic products of catabolic pathways, with amounts and types varying according to individual metabolic status [42,43]. Sialylated glycans structurally similar to HMOs, such as 3′-SLN, 6′-SLN, A-hepta, and 3′-sialyl-Lewis are also present in urine [42]. Previous research detected free sialylated glycans in urine of healthy individuals and classified them as sialylhexose and sialyllactose [44]. Although urinary glycans may originate from dietary sources such as cow milk, this is unlikely to be the case for sialyllactoses because they are found in cow milk at very low concentrations [8]. The natural presence of sialylated glycans in urine may explain why 3′-SLN and 6*'*-SLN were detected in infant urine but not in human milk [20,29,31], with the same observed for A-hepta and 3′-sialyl-Lewis [29,31]. Similarly, another study detected several glycans absent from human milk in the urine of breastfed infants, mostly at very low concentrations [30]. Those glycans may either originate from catabolic metabolic processes of glycoproteins or glycolipids or from metabolization of HMOs. Such findings underscore the complexity of urinary glycan profiles and the challenges in accurately attributing detected structures to specific sources. Future research should consider this complexity and apply analytical methods, which reliably distinguish HMOs from structurally similar glycans.

Moreover, analytical limitations must be considered when interpreting existing data. In some studies, HMOs were detected in blood and/or urine even though the infants were provided with non–HMO-fortified formula [22,33] and 2′-FL was detected in adults who did not receive 2′-FL supplements [23,24]. It is therefore uncertain whether the selected analytical methods can accurately measure and identify HMO structures such as 2′-FL. Several studies applied techniques that identify HMO structures but cannot distinguish between isomeric forms, such as LNT and LNnT [29,30]. Others suggest differences in the absorption of such isomers. For example, a study in adults supplemented with LNnT found no detectable concentrations in urine or blood [23], whereas 2 studies in infants reported the absorption of LNnT after oral intake [22,31]. However, analytical methods varied across the studies, and it remains unclear whether LNnT was consistently distinguishable from LNT. LNnT may also undergo degradation in the gut. Microbial β-galactosidase (microbial lactase) can break down type 2 HMOs and hydrolyze LNnT into lacto-*N*-triose II [45,46]. A similar mechanism may apply to human lactase, which is abundant in the intestine. Whether type 2 HMOs serve as substrates for human intestinal lactase-phlorizin hydrolase is unclear.

However, some analytical platforms used in the reviewed studies had difficulties distinguishing between HMO structural isomers, as well as between HMOs and glycan degradation products commonly found in blood and urine. For HMO analysis in biological samples, it is most important to use analytical platforms that are able to distinguish HMOs from structurally similar N-glycan and O-glycan degradation products that naturally occur due to normal cell and protein turnover and degradation. For example, 3′-SL and 6′-SL need to be separated from 3′-SLN, a degradation product of N-glycoproteins. Additionally, analytical platforms capable of providing absolute quantification of HMOs and distinguishing structural isomers with identical masses by distinct retention times are important. Conventional MS cannot differentiate such isomers without previous chromatographic separation. Similarly, common chromatographic methods such as HPLC or high-performance anion-exchange chromatography can face limitations in resolving these structures unless specialized columns and optimized conditions are employed [47,48]. Variability in analytical approaches, such as differences in sensitivity and specificity, may have contributed to inconsistencies in reported HMO presence in blood and urine across the studies. These methodological limitations may have led to misidentification or overestimation of HMOs in systemic circulation, thereby affecting the reliability of the data presented in this review.

Finally, the included studies were highly heterogeneous. Differences in study populations (eg, age and health status), number of individuals included, timing and type of HMO administration (breastfeeding and formula, supplement), as well as sampling time points and analytical methods complicate direct comparisons between existing studies and/or for specific questions, such as HMO absorption in infants compared with that in adults. This variability should be carefully considered when interpreting the presented data. As such, conclusions regarding the systemic availability of HMOs should be viewed as indicative rather than definitive.

However, our findings support the hypothesis that the physiological benefits of HMOs are not limited to their prebiotic mode of action but can also be attributed to their systemic availability and effects. A study in a murine model indicated that the systemic availability of 3′-SL after oral gavage or subcutaneous administration resulted in antiatherogenic effects, in part due to immunomodulation [49]. Preclinical studies have also demonstrated the direct effects of HMOs on immune and intestinal cells [50,51]. Finally, clinical studies have examined the impact of HMO supplements on metabolic markers in adults, showing that HMOs are safe and do not adversely affect metabolic parameters [52,53].

**FIGURE 2:**
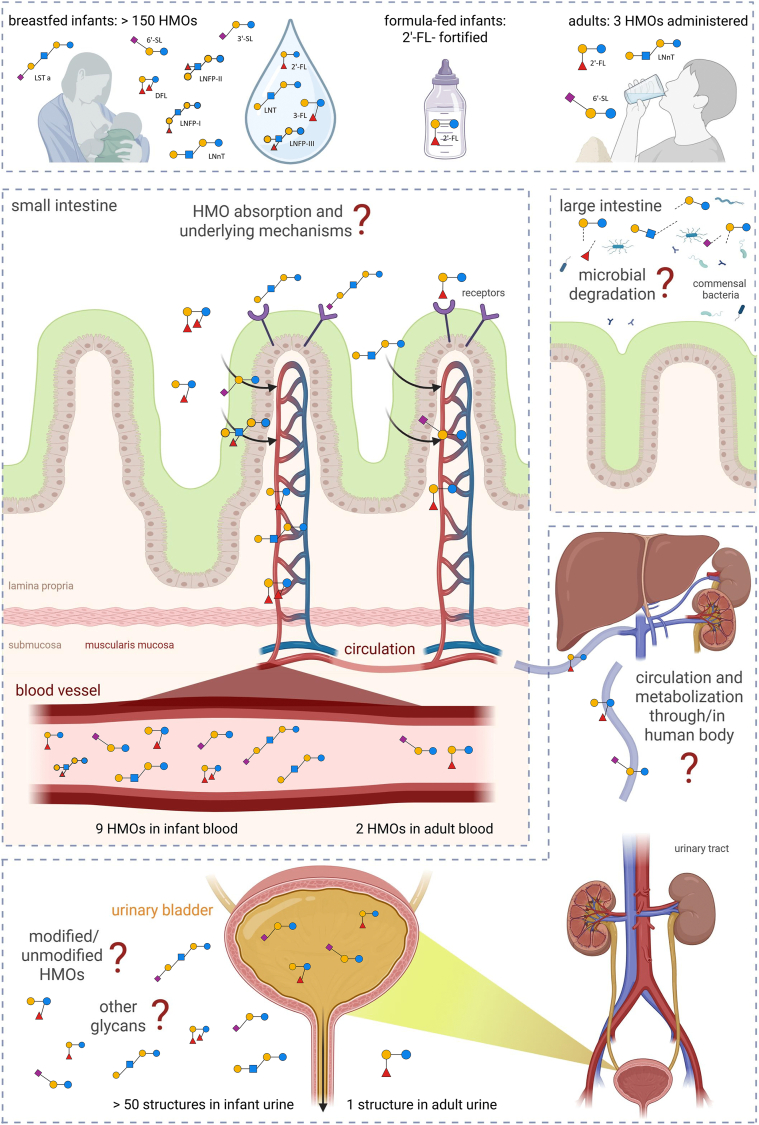
Overview of current evidence supporting human milk oligosaccharides (HMOs) presence in human blood and urine. Breastfed infants are exposed to >150 different HMOs via human milk, and 9 have been detected in their blood. In formula-fed infants, only 2′-fucosyllactose (FL) has been studied and detected in blood. In adults provided with supplements of 2′-FL, lacto-*N*-neotetraose (LNnT), or 6′-sialyllactose (SL), only 2′-FL and 6′-SL were detected in the blood, and 2′-FL in urine. More than 50 oligosaccharides were found in infant urine, although not all were clearly identified as HMOs. Current research gaps are also shown.

In conclusion, we demonstrated that different structural groups of HMOs are systemically available following oral intake in both infants and adults because they can be detected in the blood and are excreted in the urine (Figure 2). Although the presented evidence supports HMO absorption and excretion in both populations, significant research gaps remain (Box 1). Key questions, particularly regarding underlying mechanisms of absorption and pharmacokinetics, require further investigation. Controlled clinical studies with standardized protocols are necessary to obtain robust and comparable data on the systemic availability of HMOs in humans. These studies are needed to determine whether the physiological benefits of HMOs arise not only from their microbiome-dependent effects in the gastrointestinal tract but also from direct biological effects mediated through their systemic presence in human body.

BOX 1 **Research gaps in the systemic availability of HMOs in humans**

**Absorption rate.** The precise rate of HMO absorption after oral intake is unclear. Estimates range from 0.05 to 1.5% but are methodologically limited. The influence of the milk or food matrix has not been studied.

**Metabolic fate of HMOs.** HMOs in blood and urine may result from intact absorption, microbial degradation, or metabolic conversion. Their fate in human body is unknown.

**Mechanism(s) of action.** The pathways by which HMOs cross the intestinal barrier are not well understood. Specific transport mechanisms remain unclear.

**Absorption kinetics.** Kinetic data are scarce. Only 1 study (with 6′-sialyllactose) suggests rapid absorption with peak concentrations occurring after 30–60 min. Further studies are needed to determine pharmacokinetic parameters such as *C*_max_, *t*_max_, and circulation time.

**Analytical considerations.** Distinguishing HMOs from structurally similar glycans in complex biological samples requires highly specific analytical methods.

**Structure-specific differences in absorption.** It is unknown whether structurally different HMOs are absorbed at different rates. Potential differences between type 1 and type 2 HMOs remain unexplored.

Alt-text: BOX 1

HMO, human milk oligosaccharide.

## Author contributions

The authors’ responsibilities were as follows—M-CS, YBS: designed research; SS, YBS: conducted research, analyzed data, and had primary responsibility for final content; LB, M-CS, SRJ, SS, YBS: wrote the paper; and all authors: have read and approved the final manuscript.

## Data availability

Data described in the manuscript, code book, and analytic code will be made available upon request pending (eg, application and approval, payment, and other).

## Funding

The authors reported no funding received for this study.

## Conflict of interest

SRJ and YBS are employees of Novonesis, a company that produces HMOs. This affiliation did not influence data interpretation or manuscript preparation. All other authors report no conflicts of interest.
