# Supplementary material for: Systemic Availability of Human Milk Oligosaccharides in Infants and Adults: A Narrative Review
Source: Adv Nutr. 2025 Aug 6;16(9):100488. doi: 10.1016/j.advnut.2025.100488 (PMC12444163; doi:10.1016/j.advnut.2025.100488)
Supplement: Multimedia component 1 [file mmc1.pdf]

Supplementary Materials

Supplementary Table 1: Oligosaccharide concentrations/abundances in human blood and urine samples

Study	Oligosaccharides in blood: concentration/abundance	Oligosaccharides in urine: concentration/abundance
Radzanowski et al. 2013 (19)	concentration ranges:<br>2′-FL: 0–2.25 g/L[1]<br>3′-SL: 0.10–0.78 mg/L<br>6′-SL: 0.05–0.68 mg/L	not applicable
Goehring et al. 2014 (20)	mean concentrations and standard error of the mean (SEM):<br>Mean ± SEM:<br>2′-FL (breastfed):<br>1.16 ± 0.29 mg/L<br>2′-FL (breastfed and secretor (Se^+)):<br>1.66 ± 0.27 mg/L<br>2′-FL (breastfed and nonsecretor (Se^-)):<br><0.03 mg/L<br><br>not quantified for the other detected structures 3-FL and LNnT<br><br>relative fractions of the amount ingested appear to be low, 0.1% for blood	mean concentrations and standard error of the mean (SEM):<br>Mean ± SEM:<br>2′-FL (breastfed)<br>108 ± 65 mg/L<br>2′-FL (breastfed and Se^+):<br>133 ± 78 mg/L<br>2′-FL (breastfed and Se^-):<br><0.03 mg/L<br><br>6′-SL (breastfed)<br>26.1 ± 17.2 mg/L<br>6′-SL (breastfed and Se^+):<br>31.5 ± 22.4 mg/L<br>6′-SL (breastfed and Se^-):<br>8.3 ± 2.4 mg/L<br><br>not quantified for the other detected structures 3-FL, LNFP I, LNFP II, LNFP III and LNnT<br><br>relative fractions of the amount ingested appear to be low, 4% for urine
Ruhaak et al. 2014 (22)	absolute average concentrations:<br><br>6′-SL:<br>150 ng/mL in partially breastfed<br>23 ng/mL in exclusively formula-fed<br><br>LNT:<br>77 ng/mL in partially breastfed<br>7 ng/mL in exclusively formula-fed	not applicable

Marriage et al. 2015 (21)	2′-FL concentrations:<br>42 d: 0.13–1.00 mg/L<br>119 d: 0.05–0.43 mg/L<br><br>relative absorption of 2′-FL:<br>42 d: 0.05– 0.07%<br>119 d: not stated	2′-FL concentrations:<br>42 d: 3.00–35.55 mg/L<br>119 d: 2.88–19.52 mg/L<br><br>relative excretion of 2′-FL:<br>42 d: 1.26–1.50%<br>119 d: not stated
Rudloff et al. 1996 (32)	not applicable	mean excretion of neutral sugars deriving from complex oligosaccharides:<br>breastfed: 3.8 ± 2.1 mg kg/day<br>formula-fed: 2.9 ± 0.9 mg kg/day
Chaturvedi et al. 2001 (33)	not applicable	oligosaccharide levels in urine samples of breastfed infants were significantly higher than in those of formula-fed infants:<br>breastfed: 36 ± 10 nmol/mL (µmol/L)<br>formula-fed: 4 ± 1.5 nmol/mL (µmol/L)<br><br>0.5% of consumed milk oligosaccharides are absorbed and excreted into urine
De Leoz et al. 2013 (31)	not applicable	infant's urine:<br>56% non-fucosylated, neutral HMOs<br>29% fucosylated HMOs<br>13% sialylated HMOs<br>2% sialylated, fucosylated HMOs<br><br>abundance (counts per second) of oligosaccharide structures:<br>3′-SL: 370266<br>DFL: 11197<br>6′-SLN: 115044<br>3′-SLN: 227705<br>LNT: 4096560<br>LNnT: 83004<br>3′-sLe: 4368<br>LNFP II: 1060058<br>LNFP III: 315594<br>LST b: 119850<br>LNDFH I: 72956<br>LNH: 14418<br>SFLNnT: 18919<br>A-hepta: 55211<br>DFLNH a: 27489<br>MSMFLNH I: 10516<br>5130a: 29970

Dotz et al. 2015 (30)	not applicable	not quantified
Underwood et al. 2015 (29)	not applicable	mean percentages of groups of HMOs:<br>mean% (standard deviation (SD)):<br>fucosylated: 40.3 (21.4)<br>neutral: 36.3 (24.5)<br>sialylated: 20.1 (13.0)<br>fucosylated, sialylated: 3.26 (3.32)<br><br>mean percentage of oligosaccharide structures:<br>mean% (SD):<br>3-FL: 0.761 (1.65)<br>2′-FL: 8.67 (11.4)<br>DFL: 5.10 (6.64)<br>LNT/LNnT: 29.6 (25.0)<br>LNFP II: 0.779 (1.91)<br>LNFP III: 0.201 (0.406)<br>LNFP I: 4.78 (9.15)<br>LNFP V: 12.3 (13.9)<br>LNDFH I/LNDFH II: 1.22 (2.09)<br>LNH: 1.90 (3.16)<br>LNnH: 1.75 (2.44)<br>p-LNH: 0.445 (0.497)<br>A-hepta: 0.0193 (0.0670)<br>six structures (MFpLNH IV, 4120a, MFLNH III, MFLNH I, IFLNH III, and IFLNH I): 6.45 (7.63)<br>four structures (DFpLNH II, DFLNHb, DFLNHa, and DFLNHc): 3.30 (4.80)<br>TFLNH: 0.138 (0.265)<br>5130a: 0.0113 (0.0392)<br>5130b: 0.326 (0.417)<br>5130c: 0.0742 (0.156)<br>F-LNO: 0.183 (0.431)<br>six structures (DFLNO I, DFLNnO II, 5230a, DFLNnO I, and DFLNO II, 5230b): 0.274 (0.584)<br>6′-SL: 0.803 (2.78)<br>3′-SL: 4.62 (8.16)<br>6′-SLN: 2.31 (3.52)<br>3′-SLN: 1.68 (4.16)<br>3′-sLe: 0.085 (0.116)<br>LST a/b/c: 6.35 (5.39)

		F-LST c: 0.0654 (0.150)<br>DSLNT: 0.412 (0.543)<br>S-LNH: 0.484 (0.449)<br>4021a: 0.570 (1.97)<br>S-LNnH II: 0.962 (1.53)<br>4021b: 0.151 (0.284)<br>seven structures (4121a, 4121b, FS-LNH III, FS-LNH, FS-LNH I, FS-LNnH I, and FS-LNH II): 2.68 (2.73)<br>DFS-LNH: 0.183 (0.279)<br>5031a: 0.113 (0.188)<br>FS-LNO/5131a: 0.0456 (0.745)
Obermeier et al. 1999 (27)	not applicable	0.6% of the ingested ^{13}C amount appeared over 20.1 h in the infant's urine<br><br>in the 14 h after the intake of the ^{13}C bolus, the woman excreted in her urine ~1% of the applied ^{13}C bolus (data not shown)
Rudloff et al. 2012 (26)	not applicable	renal excretion of LNT and LNFP II varied between 1 and 3 mg per day<br><br>concentration in µg/mL:<br>mean (SD):<br>LNT: 7.3–21.2 (0.1–0.5)<br>LNFP II: 7.0–22.6 (0.1–1.5)
Dotz et al. 2014 (28)	not applicable	not quantified
Iribarren et al. 2021 (23)	data expressed as natural logarithm of the peak area and shown as mean (min–max):<br>2′-FL: up to 8.9 (6.2–10.9)	data expressed as natural logarithm of the peak area and shown as mean (min–max):<br>2′-FL: up to 11.3 (9.3–12.8)
Park et al. 2023 (25)	mean of peak concentration (C_{max}):<br>139.4 ng/mL at 30 min in the low-dose group<br>398.5 ng/mL at 1 h in the high-dose group	not applicable
Ko et al. 2024 (24)	data are expressed as means ± standard deviations:<br><br>2′-FL group:<br>baseline: 129.6 ± 74.4 ng/mL<br>week 6: 132.2 ± 79.6 ng/mL	not applicable

	week 12: 134.8 ± 90.4 ng/mL<br>mean (SEM): 132.2 ± 11.7 ng/mL<br><br>placebo group:<br>baseline: 102.3 ± 55.2 ng/mL<br>week 6: 84 ± 42.7 ng/mL<br>week 12: 96.6 ± 55.1 ng/mL<br>mean (SEM): 94.3 ± 12 ng/mL	

[1]This is likely to be a unit error in the publication. The actual reading was probably 0–2.25 mg/L rather than g/L.
